# Accuracy of DICOM–DICOM vs. DICOM–STL Protocols in Computer-Guided Surgery: A Human Clinical Study

**DOI:** 10.3390/jcm11092336

**Published:** 2022-04-22

**Authors:** Gianmaria D’Addazio, Edit Xhajanka, Tonino Traini, Manlio Santilli, Imena Rexhepi, Giovanna Murmura, Sergio Caputi, Bruna Sinjari

**Affiliations:** 1 Unit of Prosthodontics, Department of Innovative Technologies in Medicine and Dentistry, University “G. d’Annunzio” Chieti-Pescara, 66100 Chieti, Italy; gianmariad@gmail.com (G.D.); t.traini@unich.it (T.T.); santilliman@gmail.com (M.S.); imena.rexhepi@unich.it (I.R.); giovanna.murmura@unich.it (G.M.); scaputi@unich.it (S.C.); 2 Electron Microscopy Laboratory, University “G. d′Annunzio” Chieti-Pescara, 66100 Chieti, Italy; 3 Department of Dental Medicine, Medical University of Tirana, Rruga e Dibrës, 1001 Tirana, Albania; editxhajanka@yahoo.com

**Keywords:** dental implants, guided surgery, digital workflow, stereolithographic surgical guide, accuracy, CAD–CAM, DICOM–STL, static guided surgery

## Abstract

Guided implant surgery can enhance implant placement positioning, increasing predictability and decreasing postoperative complications., To date, the best protocol to be used for template realization is still unknown. Thus, the aim herein was to clinically compare the accuracy of two different protocols. A total of 48 implants were divided into Group A (24 implants), in which a stereolithographic template was realized using the digital imaging and communications in medicine (DICOM) data arrived from cone beam computer tomographies (CBCTs) (patients and prothesis alone), and Group B (24 implant), in which a standard intraoral stent with a standardized extraoral support was used for patients’ intraoral impressions and CBCT. The preimplant virtual planning and postsurgery CBCT images of both groups were superimposed, and differences were registered in terms of average deviations at the platform (a) and implant apex (b), mean depth change (c), and angular deviation (d). The results demonstrated that there were no statistically significant differences between groups (*p* = 0.76) for the parameters measured. However, statistically significant differences (*p* < 0.05) were found between maxillary and mandible implant surgery, as the latter showed greater accuracy. Additional studies are necessary to further reduce discrepancies between planning and surgical procedures.

## 1. Introduction

To date, the use of implants in totally edentulous patients or patients with residual dentition has allowed for safe and predictable patient rehabilitation [1,2]. Over the years, different solutions have been developed, depending on the degree of atrophy and patients’ needs [3,4,5]. The success of rehabilitations depends on various factors, such as appropriate presence of hard and soft tissues, healthy systematic conditions, macro- and microimplant morphologies, correct positioning, and maintenance over the years [6,7,8,9,10,11,12]. The use of computer-guided surgery has made it possible to simplify procedures and carry out guided prosthetic rehabilitation, also allowing immediate loading procedures [13,14,15]. In this sense, computer-guided surgery represents a valid method for treating patients with extensive rehabilitations [13,14,15]. Through detailed planning, this ensures correct three-dimensional positioning while respecting the residual bone and prosthetic position [14,15,16,17]. Several studies and literature reviews have investigated the advantages and limitations of computer-guided surgery [18,19,20,21]. The advantages include the possibility of reducing trauma and the duration of surgical interventions, avoiding errors and complications [14,15,16,22]. On the other hand, any design errors in the size of the template, and consequently the impossibility of deviating from the initial design, can fall within the limits of computer-guided static surgery [16,21,23,24,25,26]. However, errors during the various steps can cause different positioning than was planned, thus eliminating the advantages of computer-guided implant insertion. These errors are mainly attributable to mistakes made during data collection, planning, or the creation of surgical templates [18,19,20,21,24] Even if the growth of dental digital technologies has significantly reduced the errors in terms of angular deviation, apex or coronal portion of the implant, and insertion depth, minimal differences between the planned and real position of the implants till remain [21]. In this regard, cone beam computerized tomography (CBCT) and intra- or extraoral scanning play key roles [27,28]. CBCT is useful for the visualization of hard tissues [27], while intra- and extraoral scanning allow obtaining information on soft tissues and prostheses [17]. Overlaying the aforementioned collected data creates a virtual patient that can be used for implant planning procedures [13,22]. Through this workflow, it is possible to obtain resin guides for implant positioning through different production processes such as milling, rapid prototyping, stereolithography, and 3D printing [29]. However, the working protocols for the aforementioned data collection are different. In fact, it is the data collection, among other steps, that can cause discrepancies by increasing the possibility of error [30,31,32]. To date, three systems have been described for the collection and matching of patients’ data that allow, through different procedures, the overlapping of information from hard tissues (both bone and teeth) and soft tissues for prosthetic planning. These three systems are: DICOM–cast, DICOM–DICOM, and DICOM–STL (standard triangulation language) [30,31,32]. In the DICOM–cast system, a radiographic template with radiopaque markers is made from a plaster model of the patient. The cast is calibrated on a parallelometer to know exactly where the reference points were inserted. Through this technique, the surgical template is always constructed from the model to have total correspondence between the position chosen for the implants and the positions determined for the radiopaque reference points [31]. 

The DICOM–DICOM protocol, or double-scan protocol, has been widely documented in the literature and is among the most widely used methods [30]. In this case, a radiographic template (with radiopaque marks) is used to obtain two sets of DICOM data. The first is obtained from CBCT performed on the patient with the radiographic template inserted. The second ones are obtained from CBCT performed on the radiographic template only (without the patient). The two sets of data are then overlaid through the common points (radiopaque marks) in both sets of data [30].

The DICOM–STL protocol is based on overlapping between the DICOM data obtained from the patient’s CBCT and the STL data obtained from an impression (intra- or extraoral cast scanning) [32]. Common points, understood as areas visible on both the DICOM and the STL file, are used to superimpose the two sets of data. They are represented by the teeth in case of partially edentulous patients and by an extraoral stent linked with the patient’s prosthesis in the case of totally edentulous patients. Extraoral stents are radiopaque and thus visible on DICOM images. Moreover, they present a shape that can be detected by the scanner (for the STL image). For this technique, extraoral reference points of known geometric shape could be matched between CBCT and STL file [32].

Several studies have compared the actual position of the inserted implants with the planned position to understand the accuracy of the digital techniques [33]. However, to the best of the authors’ knowledge, no study has ever analyzed data coupling techniques to determine which has the greatest sensitivity and smallest margin of error during surgery. Therefore, the aim of this in vivo study was to investigate the clinical accuracy of two different protocols for data matching and the realization of surgical templates, specifically DICOM–DICOM vs. DICOM–STL. The null hypothesis was that there were no differences between the two protocols in terms of precision and accuracy.

## 2. Materials and Methods

### 2.1. Study Design

For this study, 10 patients were recruited, 5 per group. A total of 48 implants were inserted. Recruited patients were 7 men and 3 women, aged between 48 and 92 years. All patients were selected according to the inclusion and exclusion criteria presented below. None of the patients was a smoker. All patients underwent preliminary CBCT examination to establish the possibility of implant insertion.

Inclusion criteria for the present study were:

1. patients of both binary genders and all races, aged between 18 and 99 years;
2. patients for whom full supportive implant rehabilitation with multiple implants was already established;
3. patients physically able to tolerate surgical and prosthetic procedures;
4. patients who agreed to going back to the dental clinic for a follow-up visit.

The exclusion criteria were:

1. patients with active infection or severe inflammation in the areas identified for implant placement;
2. patients with a smoking habit exceeding 10 cigarettes per day;
3. patients with uncontrolled diabetes or metabolic bone disease or other uncontrolled systemic diseases;
4. patients with a history of radiotherapy treatment of the craniofacial area;
5. patients with a known gestational state;
6. patients with evidence of severe bruxism or grinding;
7. patients requiring augmentation procedures for dental implant placement and/or with buccal–lingual bone ridge dimensions of less than 5 mm.

All patients were rehabilitated with full implant supported rehabilitations performed by computer guided surgery. For all patients, data were collected, and surgical guides were constructed, according to the company’s instructions. Specifically, patients were randomly divided into two groups:

Group A: Five patients treated with mucosa-supported surgical guides made using the DICOM–DICOM protocol (or double scan protocol) (Nobel Biocare Services AG P.O. Box CH-8058 Zurich-Flughafen Switzerland).
Group B: Five patients treated with mucosa-supported surgical guides made using the DICOM–STL protocol (or extraoral stent protocol) (GEASS srl Via Madonna della Salute, 23 33050 Pozzuolo del Friuli, UD).

Data collection, template production, and clinical phases are described below. Following the indications of the producers, the data were collected in different ways. Specific surgical guides were produced for each patient with dedicated components according to the group they belonged to. Patients from both groups were rehabilitated with 4 or 6 implants depending on individual diagnostic evaluations. Specifically, each group included 2 complete rehabilitations on 6 implants and 3 complete rehabilitations on 4 implants for a total of 48 implants (24 per group).

In order to reduce the variables of the study, all interventions were performed by the same surgeon, changing only the data collection protocol and consequently the template production and implant type. In cases in which the insertion torque was greater than 35 Ncm, the implant was used for immediate loading rehabilitation [34].

### 2.2. Data Acquisition and Templates Realization

Both protocols involved the fabrication of a denture or a duplicate of the patient’s own denture in radiopaque resin.

**Figure 1:**
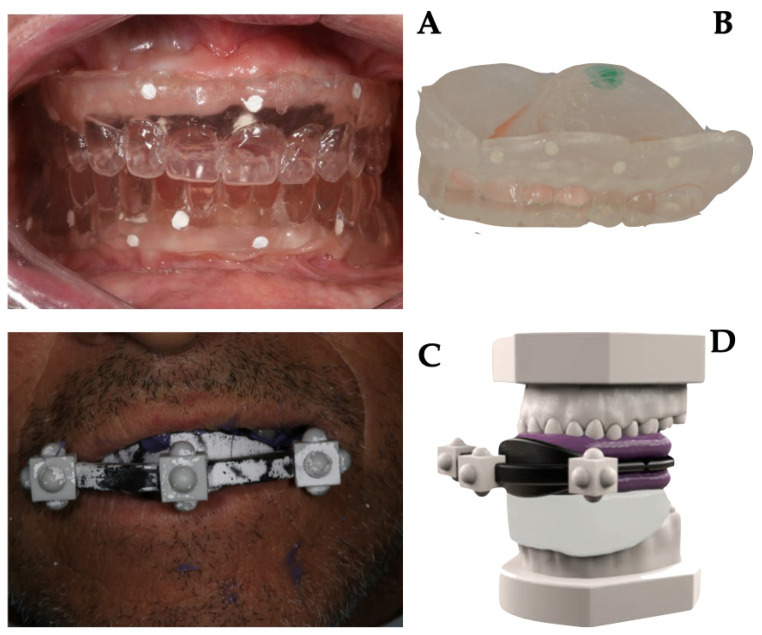
Data acquisition protocol: (**A**) intraoral position of resin duplicates of patient’s prosthesis. Gutta-percha markers were inserted into 1 mm deep niches on the prosthesis (on the vestibular and palatal–lingual sides). The patient underwent CBCT (Cone Beam Computed Tomography) scanning while wearing the denture; (**B**) a second CBCT of the denture alone was performed; (**C**) a duplicate of the prosthesis was attached to an extraoral stent with three-dimensional radiopaque marks. The whole was stabilized in the mouth with a radiolucent occlusal index. The patient underwent CBCT scanning while wearing the duplicate, stent, and stabilization index; (**D**) extraoral scans were performed using a laboratory scanner of all collected data.

Briefly, in Group A, as prescribed by the company guidelines, the gutta-percha markers (on the vestibular and palatal–lingual sides) were inserted into 1 mm deep niches on the prosthesis. Then, the prosthesis was stabilized in the patient’s mouth; the patient underwent CBCT scanning while wearing the denture, and subsequently, CBCT of the denture alone was performed. Images from the two different protocols are shown in Figure 1.

The obtained two sets of DICOM files were imported into the guided surgery software, through which the exact positions of the implants and the stabilization pins were planned in accordance with the anatomy and position of the patients’ teeth. Then, the planning data were sent to produce the stereolithographic templates.

In Group B, the duplicate of the prosthesis was attached to an extraoral stent with three-dimensional radiopaque marks and then stabilized in the mouth with a radiolucent occlusal index. The patient underwent CBCT scanning while wearing the duplicate, stent, and stabilization index. Subsequently, the following scans were performed in the dental laboratory by using a laboratory scanner (Sirona InEos X5, Dentsply Sirona Italia, Piazza dell’Indipendenza, 11, 00185 Roma RM, Italy):

- master model with radiological template and stent;
- master model at the mucosal level;
- master model without stent with only radiological template in place;
- opposite arch model.

Details of data acquisition are shown in Figure 1.

The files obtained, specifically DICOM and STL files, were imported into the guided surgery software and coupled to trace more visible landmarks. The implants were then designed in terms of length, diameter, depth, vestibulobuccal inclination, and mesiodistal inclination. After the planning phase, the template file was printed by using 3D printing (Ackuretta freeshape 120, Ackuretta technologies, 11493, Taiwan, Taipei City, Neihu District, Section 1, Neihu Rd, 322 6F).

### 2.3. Surgical Phase

All surgeries were performed by a single operator (G.D.) Prior to surgery, the surgical guide was tested on the model and in the patient’s mouth to verify correct positioning and stability. If an adequate width of keratinized gingival tissue was available at the implant site, a flapless approach was chosen; otherwise, an open-flap surgery was performed. Local anesthesia was then administered (4% articaine with adrenaline 1:100,000). The surgical guide was positioned and anchored by three bicortical bone pins. After fixation of the template, the implant sites were prepared according to the protocols provided by the manufacturers in the different groups. All drills had physical stops at the top of the drill to allow depth control. Following the standard protocol of the surgical guide system, guided milling procedures were performed, and the fixtures were inserted into the implant through the surgical guide sleeve (fully guided insertion). 

Specifically, the surgical guide was fixed in the patient’s mouth by fixation pins positioned buccally. The drills were passed through the metal sleeves in sequence. Once the length and diameter predetermined by the planning were reached, the implants were inserted by using a specific mounter for guided surgery that allowed for guided insertion of the implant until the desired position was reached. In the case of surgery with a flap, a suture with simple detached stitches was placed. In all cases, oral antibiotic therapy, anti-inflammatory therapy, and mouth rinses with chlorhexidine 0.20% for 7 days were prescribed.

### 2.4. Prosthetic Phase

All patients were rehabilitated with immediate loading fixed prostheses. Implants that did not reach a minimum insertion torque were excluded from immediate loading as previously explained [34]. The provisional prostheses were made thanks to the CAD project before implant insertion. On the surgery day, they were relined and solidified to the implant abutments. Specifically, MUAs (multiunit abutments) were placed on the implants and never removed again. Over these, temporary abutments were used for immediate loading and subsequently replaced with definitive abutments linked to the definitive prosthesis in the laboratory. The cases were finalized six months after immediate loading. Depending on the project, restorations were made in zirconia or in reinforced resin. 

### 2.5. Data Analysis 

A control CBCT was used to verify correct positioning of the implants. The same CBCT was used to verify discrepancies between the virtual plan and the actual implant position. The planning images and postoperative CBCT radiological images were overlaid by using the software’s registration algorithm aimed to verify the actual position compared with the virtual planning on the same dataset. Dataset was exported in STL format, and using the software’s best-fit algorithm, the image of the implant was then isolated and coupled with the corresponding implant project file to measure the deviation between the positions (Geomagic, Geomagic, Morrisville, NC, USA). 

**Figure 2:**
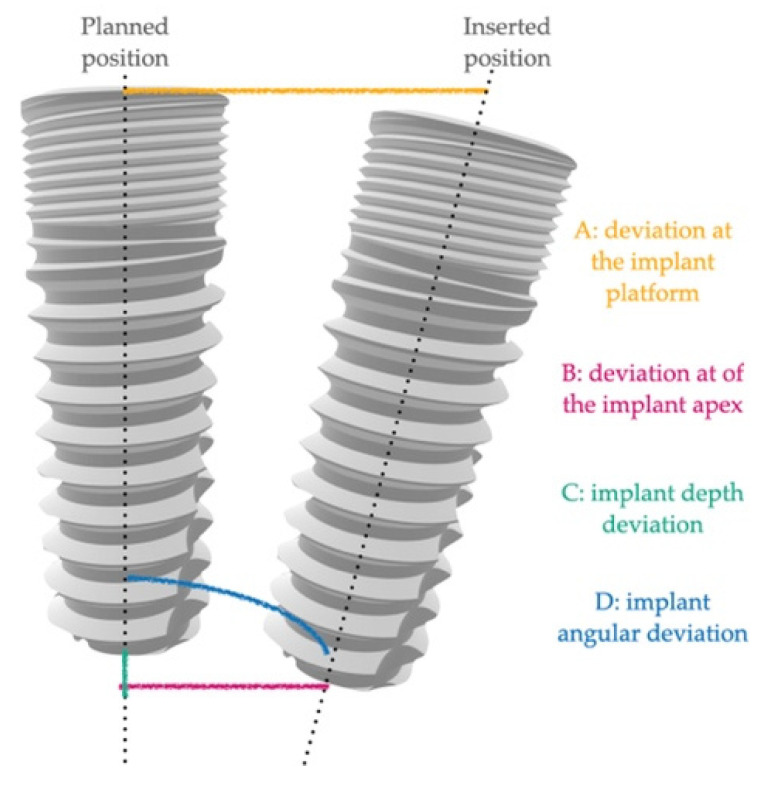
Graphical representation of the measurements. Differences between planned and inserted position were registered as: A—deviation at the implant platform, B—deviation of the implant apex, C—implant depth deviation, and D—implant angular deviation.

The following positional and angular deviations were calculated (as shown in Figure 2):

- A: deviation at the implant platform as the spatial distance between the center of the platform of the planned and positioned implants;
- B: deviation of the implant apex as the deviation at the apex level of the planned implant;
- C: implant depth deviation as the distance of the planned and positioned implants on the vertical axis;
- D: implant angular deviation the spatial angle between the planned and the positioned.

**Figure 3:**
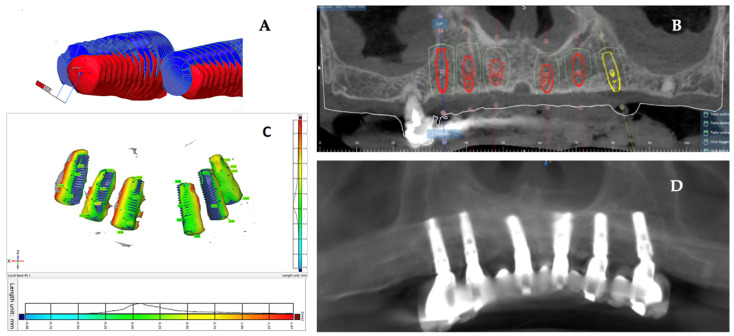
Data analysis: (**A**,**C**) superimposition of implant planning and postoperative CBCT images. It is possible to graphically see the discrepancy between the inserted and planned implants; (**B**,**D**) preoperative CBCT images with virtually inserted implants used for fabrication of surgical template; image of the postintervention control CBCT.

All measurements were repeated three times by two blinded researchers to verify the reproducibility of the record performed. Images from data analysis as shown in Figure 3.

### 2.6. Statistical Analysis

According to Vieira et al. 2013 [16], a sample size of 21 implants per group was calculated to have at the follow-up a minimum difference between the two groups. Vieira et al. reported means of 2.17 ± 0.87 and 1.42 ± 0.76 in the two groups. The value of α was determined at 0.05, while the power of the test was 0.80. The website https://clincalc.com/stats/samplesize.aspx (accessed on 3 July 2021) was used for the calculation [35]. A sample size increase by 10% was calculated to avoid patient losses at follow-up, which would invalidate the test. Therefore, 24 implants per group were selected.

Data were collected on different patients treated with different protocols to evaluate the differences between the two groups in terms of positional discrepancy. All data collected were processed with the same methodology to unify the results collected. The variables of interest were deviation at the implant platform, deviation at the apex of the implant, depth deviation, and angular deviation. Mean value, standard deviation, and range were used to describe the quantitative data. Data were analyzed with descriptive statistics to assess whether they had a normal distribution. The two-sample *t*-test and the ANOVA test (analysis of variance) were used to examine differences between the groups. Tukey tests were used to evaluate the overall significance and to perform all pairwise comparisons of the measurements between individual rehabilitation. Data analysis was performed using GraphPad version 8 statistical software. The significance level was set at *p* = 0.05. 

### 2.7. Ethical Consideration

Participants received an information sheet and provided their informed consent in accordance with the EU General Data Protection Regulation GDPR (UE) n. 2016/679 be- fore beginning the rehabilitation. The study protocol was postapproved by the Ethical Committee of the University of Medicine of Tirana on 3 November 2021. The allocation between groups was performed randomly. Additionally, patients were informed of the nature of the study and decided to freely take part in it.

## 3. Results

**Figure 4:**
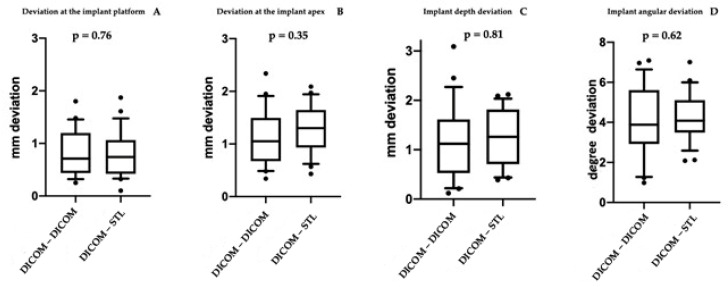
Images of the statistical comparisons between the two groups. The different positional and angular deviations analyzed did not show statistically significant differences.

**Figure 5:**
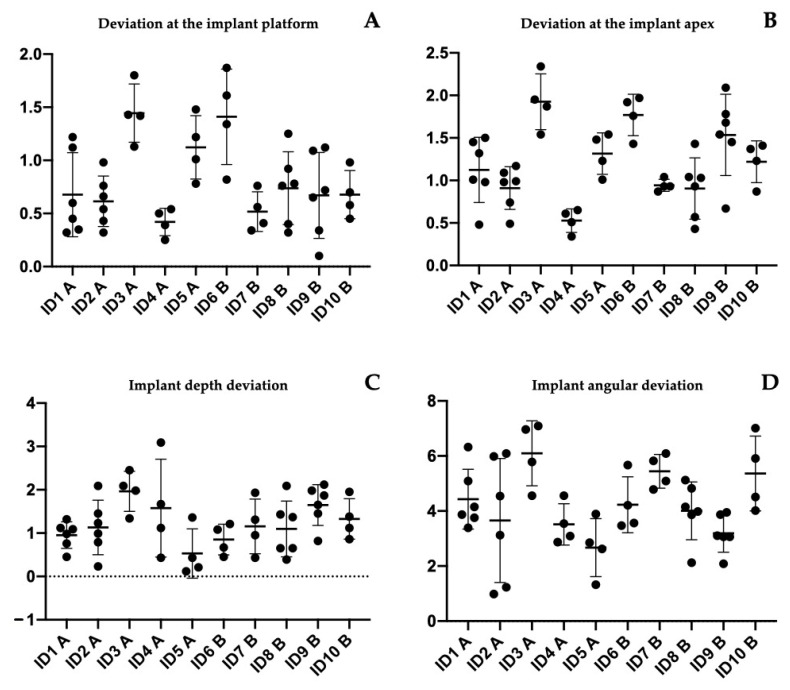
Images of statistical comparisons between the single treated patients. Tukey multiple comparison showed some statistically significant differences between patients. Specifically, as detailed in Table 4, statistically significant differences appeared between maxillary and mandibular rehabilitation. (**A**) Deviation at implant platform among all patients; (**B**) deviation at implant apex among all patients; (**C**) depth deviation among all patients; (**D**) angular deviation among all patients.

**Figure 6:**
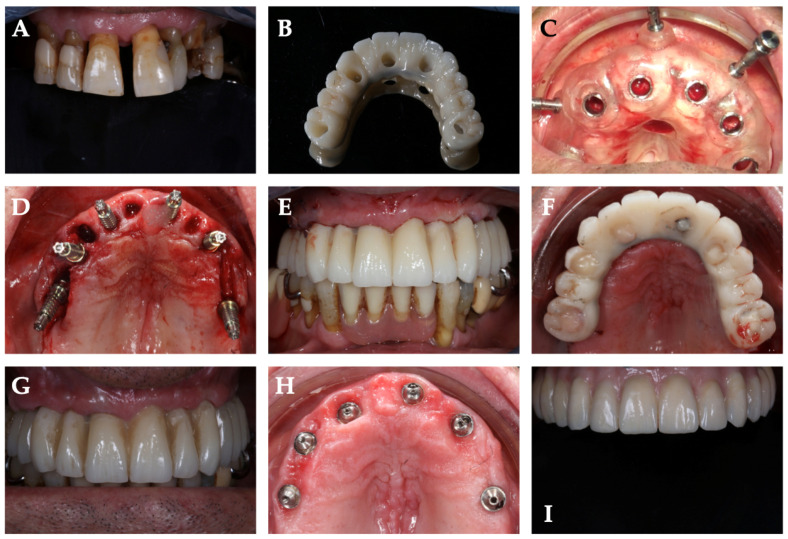
Demonstrative case from one of the treated patients: (**A**) preoperative image of hopeless dentition. Patient was rehabilitated with full arch supported by 6 implants; (**B**) CAD–CAM-milled temporary prosthesis in PMMA with metal palatal reinforcement. The provisional was perforated at the level of the prosthetic emergencies in order to be positioned and fixed after implant insertion; (**C**) surgical template positioned and fixed with 3 vestibular pins; (**D**) intraoral image with inserted implants and abutments; (**E**) frontal image of immediate loading provisional prosthesis; (**F**) occlusal image of immediate loading provisional prosthesis; (**G**) six-month control visit of the provisional restoration; (**H**) occlusal image of the abutments and soft tissues six months after implant insertion; (**I**) definitive restoration.

A total of 48 implants were placed in 10 edentulous patients. Specifically, in Group A, 16 implants were placed in the mandible and 8 in the maxilla, while in Group B, 10 implants were placed in the maxilla and 14 in the mandible. Table 1 shows the main characteristics of the patients and the inserted implants. Six patients were rehabilitated with full arches supported by four implants and four with arches supported by six implants, depending on prosthetic design and bone availability. Six months after implant placement, all included patients underwent follow-up visits to assess osseointegration before proceeding with the final prosthesis. No biological and mechanical complications were recorded. Moreover, at the one-year follow up, no intraoperative complications or implant failures were recorded, demonstrating a 100% survival rate. Table 1 shows the complications encountered during the entire study. In all rehabilitations, margins of error between the implant placed and the presurgical project were registered. However, no complications related to the placement or use of the surgical guide were observed during surgery. For all 48 implants placed, the mean deviation at the implant platform (A) was 0.803 ± 0.433 mm, while that at the apex of the implant (B) was 1.20 ± 0.484 mm. The mean change in depth (C) was 1.22 ± 0.65 mm, and the mean angular deviation (D) was 4.186 ± 1.486°. There were no statistically significant differences between the two groups *p* = 0.76 (A); *p* = 0.35 (B); *p* = 0.81 (C); *p* = 0.62 (D), accepting the null hypothesis. Table 2 and Table 3 show all differences recorded between planned and inserted implants. Figure 4 shows statistical analysis and differences between groups. Subsequently, Tukey multiple comparisons were made between individual patients. In this case, statistically significant differences (*p* < 0.05) were found between some rehabilitations. Specifically, maxillary and mandibular rehabilitations showed major differences, as mandibular restoration showed greater precision, as demonstrated in Table 4 and Figure 5. Finally, Figure 6 and Video S1 (added as Supplementary Materials) show two different clinical cases for better understanding of the study.

## 4. Discussion

Over the years, the use of CAD–CAM technologies and computer-guided surgery has enabled extensive rehabilitations to be carried out, thus reducing morbidity and postoperative discomfort and improving the predictability of restorations [17,18,21]. Thanks to these technologies, it is possible to establish the exact position of the implant in relation to the residual bone and the design of the patient’s prosthesis. Among other aspects, this reduces the surgery time and allows the prosthesis to be made properly before surgery [15]. This is all possible thanks to strict protocols established over the years, which have made it possible to standardize presurgical and surgical procedures [30]. Key factors, such as materials for surgical templates and presurgical planning, have been extensively studied to reduce the margins of error [16,21,33]. The aim of the presented study was to investigate the clinical accuracy of two different protocols for data matching and surgical templates realization. This aspect has been little investigated in the literature, and the aim was to establish which was the best protocol.

The results showed that in all placed implants, there was a margin of error between the planned and inserted implants. However, there were no statistically significant differences between the two groups. In this case, the null hypothesis of the study was accepted. Several studies have investigated the accuracy of computer-guided surgery by studying the discrepancy between implant planning and the surgical phase [18,33,36,37]. To date, in all cases, there have been differences, albeit minimal, between the planned position and surgery. A meta-analysis by Zhous et al. in 2018 [36] reported 14 in vivo studies with a mean deviation of 1.25 mm in the platform portion and an angular deviation of 4.1°. In addition, the authors reported an average deviation at the apex of 1.57 mm. The results reported herein demonstrated a reduction in the overall margins of error. This was probably related to the refinement of guided surgery protocols over the years. The same authors concluded that various factors, such as the type of template, fixation, presence/absence of residual teeth, and choice of flap can affect the level of accuracy [36]. Another review by Tahmaseb et al. in 2018 [18] reported lower margins of error, which were likely related to the inclusion of in vitro studies in the review, as the margin of error can be better controlled from the influence of factors such as mouth opening or patient reflexes that could interfere in in vivo studies [18]. Furthermore, recent studies have evaluated the accuracy of computer-guided surgery, leading to results comparable to those presented in the present study. Moreover, our results were in accordance with those of Lin et al. in 2020 [21], where an average global deviation of 0.78 mm at the implant platform and 1.28 mm at the implant apex were reported. In this case, the authors proposed a fully digital protocol, demonstrating how the margins of improvement over past years could further reduce the margin of error by exploiting the potential of digital dentistry [21].

Among the most critical factors reported in the literature, the accuracy of CBCT and possible micromovements of the mucosa-supported surgical template may have the greatest influence on planning [38]. The lack of significance in the comparison between the groups and the presence of an extremely low overall error showed that both proposed techniques, when performed rigorously, led to high performance of computer-guided surgery techniques. This translates into greater comfort for both clinician and patient. Moreover, it increases the possibility of reducing postoperative complications and shortening intervention times [15]. In the cases reported, there were no relevant complications. In two patients, the fixation screw of the prosthesis was unscrewed in the first six months. Unscrewing is one of the most common mechanical complications in implantology [39,40]. A correct tightening protocol allows reducing the occurrence of this complication [41]. Varvara et al. in 2020 demonstrated how a retightening time of 2 min led to significantly reduced preload loss [41].

In the second phase of the study, Tukey multiple comparisons were made among the individual arches treated. In this case, the results showed that the performed rehabilitations in the mandible were the most accurate. It must be remembered that the inclusion criteria allowed only patients with adequate bone availability to be considered. In this case, the factors to be taken into account to understand the various results may be the different stability of the template as well as the different bone architecture. The results presented were in accordance with the data obtained by Vieira et al. [16], which showed mean platform deviations of 2.17 mm in the maxilla and 1.42 mm in the mandible [16]. In agreement with these authors, we believe that the reduced bone density of the upper jaw can be considered the cause of the greater discrepancy with the mandibular bone. On the other hand, individual susceptibility in stabilizing surgical guidance should not be underestimated. The stop obtained from the upper jaw may reduce its movements compared with those of the mandible [16]. The use of fixation pins and intermaxillary positioning gigs make it possible to avoid this variable, leading to considering bone density as the only key element in the different result obtained between the two jaws. Vinci et al. in 2020 [33] concluded by showing a margin of error of less than 1 mm, with higher margins of error in the mandible [31]. This could be related to patient selection, which required less stringent criteria for bone availability. Ridge augmentation is necessary in case of severe atrophy [42]. Some authors described the possibility of using computer-guided surgery simultaneously with guided bone regeneration procedures to improve the predictability of the intervention or avoid such procedures [43,44]. Otherwise, bone augmentation procedures can be successfully implemented to restore volumes before guided implant insertion [42,43,44].

Within the limitations of this study, the results encourage the use of both investigated protocols in computer-guided surgery. On the other hand, the discrepancy found showed that several factors could affect this procedure, such as bone density due to anatomical differences. Therefore, the study could be expanded over time, considering more patients and evaluating further variables. In recent years, technologies have made it possible to drastically reduce the margin of error, and the results obtained show that this trend, compared with the literature of recent years, is definitely encouraging. The study, along with future improvement of technologies and protocols, could lead to the concrete elimination of this margin of error.

## Figures and Tables

**Table 1:** The table shows the main data of the treated patients. It also shows the main complications during surgical and prosthetic stages.

Patient ID	Sex	Age	Group	Implant Site	Final Torque	Immediate Loading	Six Month Complication	One Year Complication
1	M	48	A	47	45	Yes	No complication recorded	Screw loosening on one abutment. It was retightened at 15 Ncm
				46	55	Yes		
				44	60	Yes		
				34	50	Yes		
				36	35	No		
				37	55	Yes		
2	M	64	A	46	40	Yes	Screw loosening on one abutment. It was retightened at 15 Ncm	Bleeding on implants 46 and 36. Patient needed oral hygiene and instructions
				44	45	Yes		
				43	30	No		
				32	45	Yes		
				34	40	Yes		
				36	60	Yes		
3	M	64	A	14	40	Yes	No complication recorded	Occlusal adjustment
				12	50	Yes		
				22	55	Yes		
				24	40	Yes		
4	M	68	A	46	65	Yes	No complication recorded	No complication recorded
				32	55	Yes		
				34	50	Yes		
				36	55	Yes		
5	F	57	A	14	40	Yes	No complication recorded	No complication recorded
				12	30	No		
				22	55	Yes		
				24	45	Yes		
6	F	83	B	12	45	Yes	No complication recorded	Screw loosening on one abutment. It was retightened at 15 Ncm
				22	50	Yes		
				24	45	Yes		
				25	45	Yes		
7	M	92	B	44	45	Yes	No complication recorded	No complication recorded
				42	40	Yes		
				32	40	Yes		
				34	50	Yes		
8	M	69	B	16	50	Yes	Fracture of the prosthesis with immediate loading in the portion where there was a tooth in extension [26]	No complication recorded
				14	45	Yes		
				12	30	No		
				22	30	No		
				24	45	Yes		
				25	45	Yes		
9	M	69	B	46	60	Yes	Screw loosening on two abutments. They were retightened at 15 Ncm	Relining was necessary to improve the fit between the prosthesis and the gingiva
				44	55	Yes		
				43	30	No		
				32	45	Yes		
				34	50	Yes		
				36	50	Yes		
10	F	73	B	44	55	Yes	No complication recorded	No complication recorded
				42	45	Yes		
				34	50	Yes		
				32	45	Yes		

**Table 2:** Deviation measured in patients of group A.

Patient ID	Implant Position	Deviation at the Implant Platform (A)	Deviation at the Implant Apex (B)	Implant Depth Deviation (C)	Implant Angular Deviation (D)
1 (A)	47	1.22	1.5	1.09	3.39
	46	0.45	0.98	1.12	4.15
	44	0.32	0.48	0.98	3.87
	34	0.6	1.01	1.32	6.32
	36	0.35	1.45	0.45	5.09
	37	1.12	1.32	0.76	3.76
2 (A)	46	0.98	1.17	0.23	3.12
	44	0.43	0.49	1.23	4.54
	43	0.66	0.98	2.09	5.98
	32	0.54	0.74	1.45	6.09
	34	0.76	1.09	0.79	0.98
	36	0.32	0.99	0.99	1.23
3 (A)	14	1.43	2.34	1.34	6.96
	12	1.13	1.54	1.98	4.56
	22	1.42	1.87	2.09	5.78
	24	1.8	1.95	2.45	7.09
4 (A)	46	0.25	0.34	3.09	2.87
	32	0.54	0.65	1.12	4.56
	34	0.39	0.51	0.43	3.09
	36	0.5	0.61	1.67	3.54
5 (A)	14	1.01	1.23	0.21	3.89
	12	1.22	1.48	0.12	2.62
	22	1.48	1.54	0.43	1.32
	24	0.78	1.01	1.36	2.85
**Mean**		**0.8208 mm**	**1.1362 mm**	**1.1995 mm**	**4.0687°**
**St. Dev**		**0.4449 mm**	**0.5114 mm**	**0.7514 mm**	**1.7184°**

**Table 3:** Deviation measured in patients of group B.

Patient ID	Implant Position	Deviation at the Implant Platform	Deviation at the Implant Apex	Implant Depth Deviation	Implant Angular Deviation
6 (B)	12	1.34	1.43	1.21	3.56
	22	1.61	1.92	1.08	3.47
	24	1.87	1.97	0.45	5.67
	25	0.82	1.76	0.67	4.21
7 (B)	44	0.34	0.87	0.95	4.78
	42	0.41	0.93	1.31	6.09
	32	0.76	1.04	0.43	5.82
	34	0.56	0.93	1.93	5.09
8 (B)	16	0.76	0.93	0.65	3.86
	14	0.92	1.04	1.43	4.15
	12	0.32	0.43	1.37	3.98
	22	0.4	0.57	0.65	5.12
	24	0.78	1.03	0.39	4.82
	25	1.25	1.43	2.09	2.12
9 (B)	46	1.12	1.78	2.12	3.06
	44	1.09	2.09	1.87	3.87
	43	0.72	1.68	0.82	3.1
	32	0.1	0.67	1.45	3.06
	34	0.34	1.54	1.98	2.08
	36	0.65	1.45	1.65	3.95
10 (B)	44	0.45	0.87	1.38	4.02
	42	0.98	1.41	1.95	4.51
	34	0.7	1.23	1.12	5.91
	32	0.58	1.37	0.86	7.01
**Mean**		**0.7862 mm**	**1.2654 mm**	**1.2420 mm**	**4.3045°**
**St. Dev**		**0.4299 mm**	**0.4580 mm**	**0.5587 mm**	**1.2390°**

**Table 4:** Tukey multiple comparisons between patients. The only significant results are reported here. In all cases, differences emerged between maxillary and mandibula rehabilitation.

Tukey’s Multiple Comparisons Test	Mean Diff.	95.00% CI of Diff.	Sig.	Adjusted *p* Value
**deviation at the implant platform (A)**				
ID1 A vs. ID3 A	−0.7683	−1.460 to −0.07632	Yes	0.0195
ID1 A vs. ID6 B	−0.7333	−1.425 to −0.04132	Yes	0.0303
ID2 A vs. ID3 A	−0.83	−1.522 to −0.1380	Yes	0.0087
ID2 A vs. ID6 B	−0.795	−1.487 to −0.1030	Yes	0.0138
ID3 A vs. ID4 A	1.025	0.2669 to 1.783	Yes	0.002
ID3 A vs. ID7 B	0.9275	0.1694 to 1.686	Yes	0.0069
ID3 A vs. ID9 B	0.775	0.08299 to 1.467	Yes	0.0179
ID3 A vs. ID10 B	0.7675	0.009440 to 1.526	Yes	0.0451
ID4 A vs. ID6 B	−0.99	−1.748 to −0.2319	Yes	0.0032
ID6 B vs. ID7 B	0.8925	0.1344 to 1.651	Yes	0.0107
ID6 B vs. ID9 B	0.74	0.04799 to 1.432	Yes	0.0279
**deviation at the implant apex (B)**				
ID1 A vs. ID3 A	−0.8017	−1.485 to −0.1183	Yes	0.0111
ID2 A vs. ID3 A	−1.015	−1.698 to −0.3316	Yes	0.0005
ID2 A vs. ID6 B	−0.86	−1.543 to −0.1766	Yes	0.005
ID3 A vs. ID4 A	1.398	0.6489 to 2.146	Yes	<0.0001
ID3 A vs. ID7 B	0.9825	0.2339 to 1.731	Yes	0.003
ID4 A vs. ID5 A	−0.7875	−1.536 to −0.03890	Yes	0.0324
ID4 A vs. ID6 B	−1.243	−1.991 to −0.4939	Yes	<0.0001
ID6 B vs. ID7 B	0.8275	0.07890 to 1.576	Yes	0.0204
ID8 B vs. ID9 B	−0.63	−1.241 to −0.01877	Yes	0.0388
**Implant angular deviation (D)**				
ID3 A vs. ID9 B	2.911	0.2288 to 5.593	Yes	0.0243

## Data Availability

Not applicable.

## Supplementary Materials

The following are available online at https://zenodo.org/record/6273727#.Yhh_Ay9aaEc, (accessed on 21 April 2022), Video S1: Guided surgery and immediate loading restoration.
